# Correlation Between Muscle Structures and Electrical Properties of the Tibialis Anterior in Subacute Stroke Survivors: A Pilot Study

**DOI:** 10.3389/fnins.2019.01270

**Published:** 2019-11-26

**Authors:** Chengpeng Hu, Huijing Hu, Xiaopeng Mai, Wai Leung Ambrose Lo, Le Li

**Affiliations:** ^1^Department of Rehabilitation Medicine, The First Affiliated Hospital, Sun Yat-sen University, Guangzhou, China; ^2^Guangdong Industrial Injury Rehabilitation Center, Guangzhou, China

**Keywords:** correlation study, electrical impedance, stroke, tibialis anterior, ultrasonography

## Abstract

Electrical impedance myography (EIM) is a non-invasive diagnostic tool that assesses the muscle inherent properties, whereas ultrasonography can assess the alteration in muscle architecture. This study aimed to combine EIM with ultrasonography to assess the changes of the tibialis anterior (TA) muscle properties during passive plantar/dorsiflexion in stroke survivors. Fifteen patients with subacute stroke were recruited. The muscle structures were simultaneously assessed by EIM and ultrasonography at five different extension angles (−10°, 0°, 10°, 20°, and 30°) of the ankle joint. The EIM parameters measured were resistance (*R*), reactance (*X*), and phase angle (θ). The parameters recorded by ultrasonography were pennation angle (PA), muscle thickness (MT), and fascicle length (FL). Two-way repeated ANOVA was performed to compare the differences between the affected and unaffected sides as well as the parameters that changed with joint angle. Linear correlation analysis was conducted to assess the association between muscle parameters and clinical scores. The results showed that as the ankle was passively plantarflexed, the θ (*P* = 0.003) and PA (*P* < 0.001) values decreased, and the *X* (*P* < 0.001), *R* (*P* < 0.001), and FL (*P* < 0.001) values increased. Significant correlations were found between the FL and *R* values (*r* = 0.615, *P* = 0.015), MT and *R* values (*r* = 0.522, *P* = 0.046), and FL and θ values (*r* = 0.561, *P* = 0.03), as well as between the PA and the Fugl–Meyer Assessment of Lower Extremity score (*r* = 0.615, *P* = 0.015), the *R* and the Modified Ashworth Scale (MAS) score (*r* = 0.58, *P* = 0.023), and the PA and the manual muscle testing (MMT) score (*r* = −0.575, *P* = 0.025). This study demonstrated a correlation between the EIM and the ultrasonography parameters at different joint angles. Therefore, both methods could jointly be applied in patients with stroke to detect changes in the muscle inherent properties and muscle architecture. This could assist clinicians to quantitatively evaluate the muscle condition in people with subacute stroke. The study was registered on the Chinese Clinical Trial Registry (trial registration number: ChiCTR-IOR-17012299, http://www.chictr.org.cn/showprojen.aspx?proj=19818).

**Clinical Trial Registration Number:** ChiCTR-IOR-17012299.

## Introduction

Stroke brings considerable public health and commercial burden to the society and was proposed as one the of core diseases in demand of prevention and management in the global consensus (Feigin et al., 2017). It is known that muscle denervation, disuse, and atrophy contribute to muscle fiber loss and intramuscular fat infiltration in patients with stroke, which is related to muscle weakness (Scherbakov et al., 2015; Berenpas et al., 2017). Muscle architecture plays a crucial role in muscle properties and is closely related to the joint function. The progressive decrease in muscle fiber size and motor units quantity lead to the shrinking of the muscle cross-sectional area, which ultimately contributes to the reduction in muscle strength, and difficulty in performing functional activities (Sions et al., 2012). Therefore, quantitative evaluation techniques of muscle properties changes are greatly needed in stroke clinics to monitor disease progression and evaluate the effectiveness of intervention. Electrical impedance myography (EIM) is a non-invasive and easy-to-use technique to assess the muscle inherent properties. By sending a small electrical current to the tissue and detecting the surface voltage, the parameters of resistance (*R*), reactance (*X*), and phase angle (θ) of the muscle tissue can be deduced. These parameters correspond to the muscle extracellular and intracellular fluids, the cell membrane integrity, and tissue interfaces, respectively (Shiffman et al., 1999; Mulasi et al., 2015). They are considered as biomarkers that can be used to assess neuromuscular disease progression and response to therapy (Rutkove et al., 2002). In comparison with other muscle property assessment techniques such as magnetic resonance imaging and electromyography, EIM has less requirement on patients’ cooperation or subjective judgments, and has shorter time on performing the measurement and data analysis (Rutkove and Sanchez, 2019). However, as an indirect measurement method, EIM itself could not record other architecture information such as the muscle fiber arrangement. Further understanding of the clinical application of the EIM parameters is still needed.

Ultrasonography is widely used to obtain visual information on the muscle architecture. Its operation is simple and can be combined with other instruments, such as a dynamometer. Muscle architecture measured by ultrasonography usually refers to the pennation angle (PA), muscle thickness (MT), and fascicle length (FL) (Legerlotz et al., 2010). Applying ultrasonography to assess the muscle architecture has been demonstrated to be a reliable technique in the field of neuromusculoskeletal disorders (Raj et al., 2012; Cho et al., 2014, 2017). Previous studies with ultrasonography in patients with stroke revealed an altered muscle architecture, including the PA, MT, and FL on the affected side (Gao et al., 2009). Heckmatt et al. (1980, 1982) applied ultrasound to quantitatively evaluate muscle echo intensity with four grades in the transverse plane of the muscle to reflect muscle fibrosis grade in patients with Duchenne muscular disease. Increased echo intensity is usually regarded as a reflection of myofiber loss (Grimm et al., 2013). But it is still an indirect measurement and could not provide information about the muscle composition and inherent properties. As EIM and ultrasound are totally two different technologies that do not interfere with each other, the question is whether these two could be combined to evaluate muscle changes after stroke.

The reduced innervation of the tibialis anterior (TA) plays a major role in limiting ankle plantar flexion and inversion poststroke (Daniilidis et al., 2017). The dysfunction of the TA combined with substantial triceps surae spasticity (Jakubowski et al., 2017) leads to drop foot, which is described as the inability to perform active dorsiflexion during the swing phase of the gait cycle (Fatone et al., 2009) and compensate with an excessive extension of the hip and knee joints to avoid toe strike. Drop foot increases time consumption and the risk of falls during walking (Franceschini et al., 2003; Kottink et al., 2004). Several studies explored the muscle architecture alteration in the TA and the medial head of the gastrocnemius (MG) muscles post interventions (Liu et al., 2014; Hwang et al., 2015). Other literature also reported alteration of the TA (Liu et al., 2014) and MG (Gao et al., 2009) muscles when ankle or knee joint angles were passively adjusted. However, the underpinning mechanism of the muscle-joint-dependent changes after stroke is not fully understood. Information on the muscle structural changes in relation to the joint angles may provide additional understanding on the mechanism of the muscle function and structure alteration in neuromuscular disorders. The quantitative analysis of the muscle properties of the TA is essential to understand the ankle joint dorsiflexion function recovery. A better interpretation of the changes in the TA muscle inherent properties and their association with its functional activities and the ankle joint biomechanical capabilities may provide further insight into the contributing factors for muscle weakness.

The aim of this study was to explore the change in the muscle properties in patients with stroke by combining EIM with ultrasonography. The analysis of the association between EIM, ultrasonography, and a clinical scale may provide further insights on the relationship between muscle properties, and clinical functions. This study also investigated the alteration of EIM and ultrasound parameters during passive ankle dorsiflexion, which was known as a joint-angle-dependence phenomenon. We hypothesized that ultrasound and EIM could differentiate the changes as the TA muscle was passively stretched. Ultrasound and EIM parameters correlate with each other in detecting the alteration of the muscle structure and component properties in people with subacute stroke.

## Materials and Methods

### Study Design

This study was a self-controlled, cross-sectional pilot study. Muscle structure and electrical properties of the TA were recorded by ultrasound and EIM, respectively. This prospective study was conducted at the Department of Rehabilitation of the First Affiliated Hospital of Sun Yat-sen University, China. from May to December 2018. This study was approved by the Human Subjects Ethics Subcommittee of the First Affiliated Hospital of Sun Yat-sen University. The study was registered on the Chinese Clinical Trial Registry (trial registration number ChiCTR-IOR-17012299). All participants provided written informed consent prior to taking part in the experiment. All procedures were performed in accordance with the Declaration of Helsinki.

### Participant

We recruited 15 patients with subacute stroke (3 females and 12 males; mean age, 56.4 ± 13.17 years). The inclusion criteria were as follows: (1) stroke with unilateral hemiparesis lesions confirmed by magnetic resonance imaging or computed tomography; (2) hemiparesis within 6 months after a stroke; (3) available ranges of motion at the bilateral ankle joints between 10° of passive dorsiflexion and 30° of passive plantar flexion; (4) ability to follow instructions to complete the required tasks; (5) no history of a surgical procedure or traumatic injuries of the ankle joint; and (6) absence of unexpected medical complications that would require medical attention at the time of data collection, such as epilepsy, myocardial infarction, and aortic dissection. The exclusion criteria were as follows: (1) patients with Modified Ashworth Scale (MAS) score of 4 during ankle dorsiflexion when the knee is extended; (2) patients who were not willing to participate; and (3) patients who were not able to concentrate or be cooperative.

### Apparatus and Procedures

This study utilized a cross-sectional design with the combination of EIM and ultrasonography assessment of the bilateral TA muscles.

### Isometric Dynamometer

**FIGURE 1:**
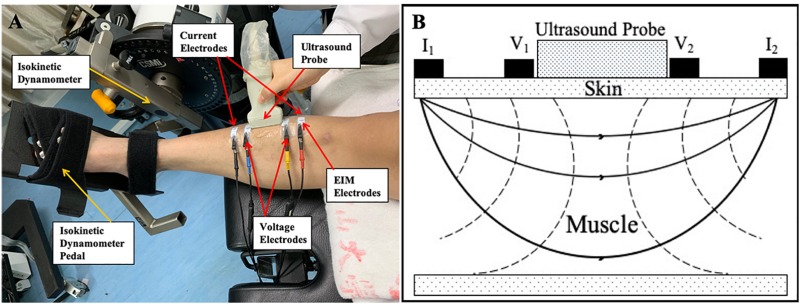
**(A)** One male subject was relaxed and in supine position on an instrumented bed with foot fixed on the isokinetic dynamometer pedal. One pair of current electrodes (red and black) and one pair of voltage electrodes (yellow and blue) were linearly arranged along the surface of the TA on the muscle belly. An ultrasound probe was placed between voltage current electrodes. **(B)** Demonstration of positions of EIM parameters and ultrasound probe. I_1_ and I_2_ were a pair of current electrodes of EIM. V_1_ and V_2_ were a pair of voltage electrodes of EIM. An ultrasound probe was placed between these two pairs of electrodes. TA, tibialis anterior; EIM, electrical impedance myography.

Participants were supine on an instrumented bed with the ankles resting in neutral position on the pedal of an isokinetic dynamometer (HUMAC NORM, Computer Sports Medicine, Inc., MA, United States). The center of the ankle joint was on the same level as the rotation axis of the device, and the ankle dorsiflexion/plantar-flexion axis was aligned with the vertical axis of the dynamometer (Figure 1A). The range of motion (ROM) was set between 10° of dorsiflexion and 30° of plantar flexion on the dynamometer. Five points for measurements were chosen: (1) dorsiflexion of 10° (−10°); (2) neutral position (0°); (3) plantar flexion of 10°; (4) plantar flexion of 20°; and (5) plantar flexion of 30°. At each joint angle, EIM and ultrasound assessment were performed simultaneously. The sequence of the measuring angle was randomized, and all measurements were recorded from the bilateral TA muscles.

### Electrical Impedance Myography

EIM (Imp SFB7 Impedimed, Inc., Sydney, NSW, Australia) assessment was performed at the muscle belly of the TA muscle. The central point of the muscle belly of the TA was marked as one third of the distance from the proximal site of the fibular head to the center of the medial malleolus. Saline was applied to moisten the skin before each test. Four electrodes, one pair of current electrodes on the outer regions and an inner pair of voltage electrodes, were arranged linearly parallel to the muscle fiber direction. The distance of the inner two voltage electrodes was 40 mm, and that of the outer two current electrodes was 90 mm (Rutkove et al., 2005). Each pair of electrodes was distributed symmetrically along the marked center point. The dimension of each electrode was 2.5 cm × 1 cm (Figure 1). Three measurements were recorded at each joint angle. The mean value of the three measurements was used for statistical analysis.

The data recorded by the EIM device were exported to Bioimp Software for offline analysis. The impedance parameters, *R*, *X*, and phase angle [θ = arctan(*X*/*R*)], were recorded across multiple frequencies of between 5 kHz and 1,000 kHz. The *R*, *X*, and θ values were recorded at the frequency of 100 kHz to avoid an interference with the high impedance from the electrode–skin contact surface at frequencies lower than 50 kHz (Li L. et al., 2017).

### Ultrasonography Measurement

**FIGURE 2:**
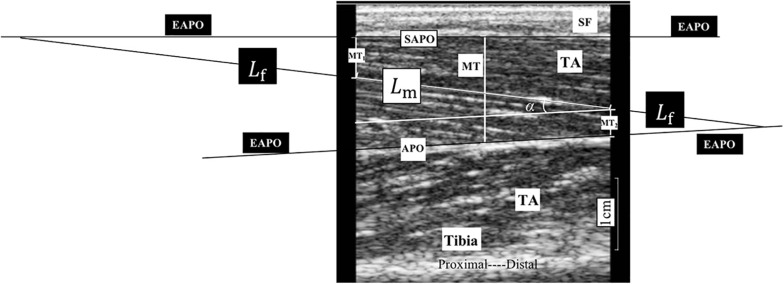
A typical ultrasonography image of non-paretic tibialis anterior (TA) from a participant during assessment. MT_1_ and MT_2_ are the distance of the visible fiber distal end point to the superficial aponeurosis and the distance of the visible fiber proximal end to the aponeurosis, respectively; and α is the pennation angle (PA). We considered the middle thickness of captured image as the muscle thickness (MT) of the TA, so we measured, respectively, the thickness of both left and right edges of the TA in the image and take the average value of these two measurements as MT for statistical analysis. APO, aponeurosis; EAPO, extension of the visible aponeurosis; SAPO, superficial aponeurosis; *L*_f_, entire estimated muscle fascicle length; *L*_m_, visible part of the muscle fiber in the image; MT, muscle thickness; SF, subcutaneous fat; TA, tibialis anterior.

Ultrasonography was performed using a B-mode ultrasonography scanner (DP6600, Mindray Inc., China) with a 7.5-MHz, 38-mm probe (imaging resolution, 0.3 mm; frame rate, 25/s). During the examination, the ultrasound probe was placed perpendicularly to the muscle fibers of the TA muscle between the two inner voltage electrodes (Figure 1). Coupling gel was applied before the examination to enhance the ultrasound conduction between the probe and the skin surface. Three ultrasound measurements were recorded at each ankle joint angle, and the average was calculated for statistical analysis. The images recorded at each of the measuring joint angles were saved for the calculation of the muscle morphology parameters. A typical ultrasound image of the TA muscle is shown in Figure 2. The PA, MT, and FL were calculated from the ultrasound image. The PA was directly measured from the image. The MT was calculated as the average of the values obtained between the two edges of the TA muscle in the image. Muscle FL was estimated using a trigonometry method, which assumed a linear continuation of the muscle fascicle (Reeves and Narici, 2003; Li et al., 2007). Equation 1 was used to derive the muscle FL:

(1) $$L_{\text{f}}=L_{\text{m}}+{\text{MT}}_{1}/\sin\alpha +{\text{MT}}_{2}/\sin\alpha$$

where *L*_f_ is the entire estimated muscle FL; *L*_m_ is the visible part of the muscle fiber; MT_1_ and MT_2_ are the distances from the fiber distal endpoint to the superficial aponeurosis and from the fiber proximal endpoint to the aponeurosis, respectively; and α is the PA (Figure 2).

### Clinical Assessments

Fugl–Meyer assessment of lower extremity (FMA-LE), MAS, and manual muscle testing (MMT) were performed by a physical therapist who was blinded to the EIM and ultrasonography results. MAS and passive ROM of ankle joint were measured when the subjects were supine on the instrumented bed of the dynamometer with the knee fully extended.

### Data Processing

All parameters (*R*, *X*, θ, PA, MT, and FL) recorded at each ankle joint angle were averaged to reflect the average alteration in the passively stretched muscle. This average value was labeled as *A* value. The difference in the *A* value between the affected and unaffected sides (dA value) was integrated (Equation 2) for the correlation analysis with the clinical scales, as follows:

(2) $$A_{x}=\frac{x_{-10{}^{\circ }}+x_{0{}^{\circ }}+x_{10{}^{\circ }}+x_{20{}^{\circ }}+x_{30{}^{\circ }}}{5}$$

The *A* value is the average value of the five ankle joint angles, and *x* represents the individual parameters, including the *R*, *X*, θ, PA, MT, and FL values. Besides the raw data obtained directly, the slope of all parameters at each ankle joint angle was also calculated by applying the data measured at the five angle points in a linear regression analysis. This slope value was labeled as *S* value. The *S* value can reflect the muscle property variation trend while being passively stretched. In addition, we calculated the difference in the *S* value between the affected and unaffected sides (dS value) for the correlation analysis.

### Statistical Analysis

The mean and standard deviation of all parameters were analyzed. Two-way repeated ANOVA with *post hoc* test was used to compare the *R*, *X*, θ, PA, MT, and FL values (angle and side) within the bilateral TA muscles at the different ankle joint angles (i.e., −10°, 0°, 10°, 20°, and 30°). If there was a significant interaction between the angle factor and the side factor in the two-way ANOVA, the paired-sample test was used to compare the differences between the affected and unaffected sides at each joint angle point. The slope value was compared between the affected and unaffected sides using the paired-sample test. The paired-sample *t* test was performed if the dataset was normally distributed (Li X. et al., 2017), whereas the Wilcoxon matched-pairs signed ranks sum test was performed for the non-normally distributed dataset. The correlation analyses were performed between the dS values of the EIM parameters (dSR, dSX, and dSθ) and the dS values of the ultrasound parameters (dSPA, dSFL, and dSMT), as well as between the dA values of the EIM and ultrasonography parameters (dAR, dAX, dAθ, dAPA, dAFL, and dAMT) and the clinical scale scores. Pearson’s correlation analysis was employed for the normally distributed dataset, whereas Spearman’s correlation analysis was employed for the non-normally distributed dataset. Data normality was verified using the Shapiro–Wilk test. A two-tailed *P* ≤ 0.05 was considered statistically significant for all calculations. All statistical analyses were conducted using the SPSS software, version 23 (IBM Inc., WA, United States).

## Results

Fifteen patients with subacute stroke (3 females and 12 males; mean age, 56.4 ± 13.17 years) were recruited in the study. Table 1 shows the demographic and clinical information for the cohort.

**TABLE 1:** Clinical characteristics of the sample population.

**ID**	**Age range (years)**	**Gender**	**Type of stroke**	**Duration (m)**	**Paresis side**	**MAS (dorsiflexion)**	**MMT (dorsiflexion)**	**FMA-LE**
1	41–45	Male	Ischemic	1	Left	1	3	24
2	61–65	Male	Ischemic	3	Right	0	5	26
3	30–35	Male	Ischemic	2	Right	0	4	28
4	51–55	Male	Hemorrhagic	4	Left	3	4	21
5	60–65	Male	Hemorrhagic	5	Left	0	4	27
6	56–60	Female	Ischemic	1	Left	0	2	19
7	46–50	Male	Ischemic	3	Left	1	4	23
8	66–70	Female	Ischemic	1	Left	0	4	30
9	30–35	Male	Hemorrhagic	1	Left	1	2	15
10	56–60	Male	Ischemic	1	Left	1	1	18
11	56–60	Male	Ischemic	3	Right	1	5	25
12	66–70	Male	Ischemic	4	Right	2	3	23
13	61–65	Male	Ischemic	1	Left	0	4	14
14	76–80	Female	Ischemic	3	Right	0	2	19
15	66–70	Male	Ischemic	1	Right	0	5	25

*MAS, modified ashworth scale; MMT, manual muscle test; FMA-LE, Fugl–Meyer assessment of lower extremity.*

### EIM Parameters

**FIGURE 3:**
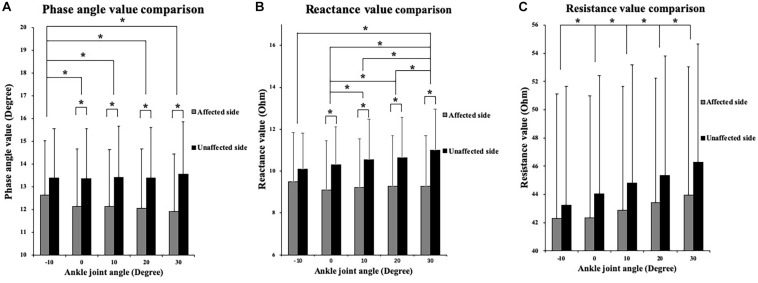
Two-way repeated ANOVA with *post hoc* analysis results of EIM parameters. **(A)** Phase angle (θ) value comparison among five ankle joint angles. **(B)** Reactance (*X*) value comparison among five ankle joint angles. **(C)** Resistance (*R*) value comparison among five ankle joint angles, and significant difference was revealed between random two groups of ankle joint angles. The *Y* axis of *A*, *B*, and *C* did not start from 0 to demonstrate the variation trend of parameters versus joint angle as well as comparison between three parameters. ^∗^*P* ≤ 0.05. ANOVA, analysis of variation; θ, phase angle; *X*, reactance; *R*, resistance; EIM, electrical impedance myography.

The two-way ANOVA revealed a significant main effect of the angle factor for the θ (*P* = 0.003), *R* (*P* < 0.001), and *X* (*P* < 0.001). A significant main effect of the side factor was found for the θ (*P* = 0.044) and *X* (*P* = 0.021). A significant angle–side interaction effect was found for the θ (*P* < 0.001), *R* (*P* = 0.005), and *X* (*P* < 0.001). As the ankle joint plantar flexion angle increased, the θ value decreased, and the *R* and *X* values increased. Lower *X* and θ values were found on the affected side than on the unaffected side for all five angle points. In the paired-sample test comparisons between the affected and unaffected sides, the θ values were significantly lower in the affected TA muscle at the angle points of 0° (*P* = 0.023), 10° (*P* = 0.015), 20° (*P* = 0.011), and 30° (*P* = 0.005) than in the unaffected side. In addition, significantly lower *X* values were found in the affected TA muscle at 0° (*P* = 0.027), 10° (*P* = 0.016), 20° (*P* = 0.015), and 30° (*P* = 0.006). No significant difference was observed in the *R* values between the affected and unaffected sides (Figure 3).

### Ultrasonography Parameters

**FIGURE 4:**
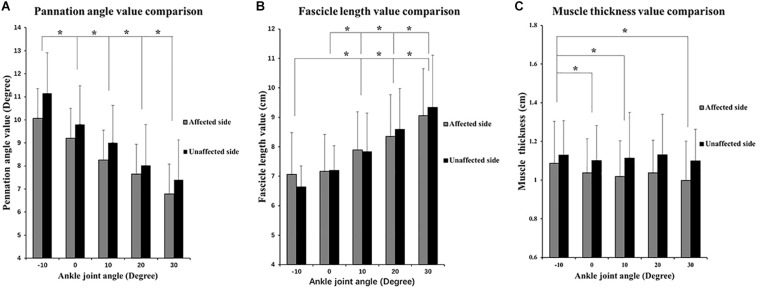
Two-way repeated ANOVA with *post hoc* analysis results of ultrasonography parameters. **(A)** PA values comparisons among five ankle joint angles. **(B)** Fascicle length (FL) values comparison among five ankle joint angles, and significant difference was revealed between random two groups of ankle joint angles. **(C)** MT value comparison among five ankle joint angles. *Y* axis of *A*, *B*, and *C* did not start from 0 to demonstrate the variation trend of parameters versus joint angle as well as comparison between three parameters. ^∗^*P* ≤ 0.05.

**FIGURE 5:**
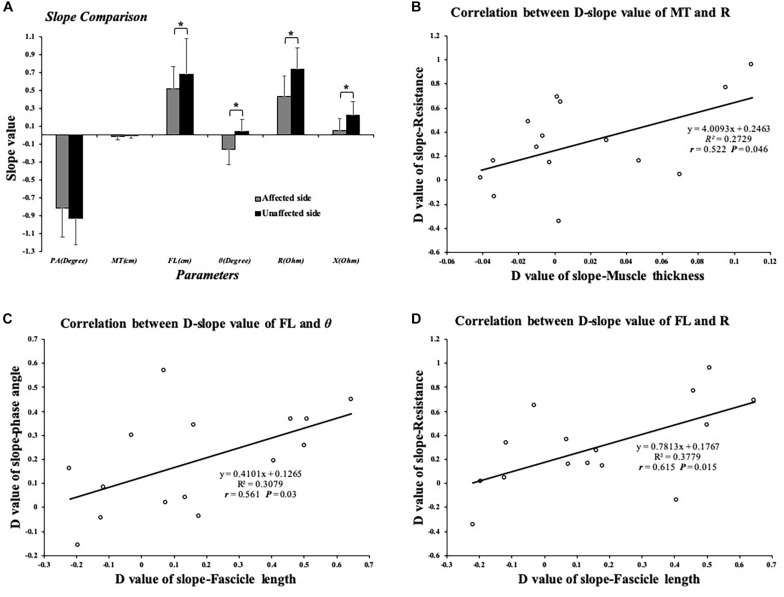
**(A)** Comparison of slope value of EIM and ultrasound parameters; significant difference was indicated in SFL, SR, SX, and Sθ between affected and unaffected sides. **(B)** Significant correlation between dSMT and dSR values. **(C)** Significant correlation between dSFL and dSθ. **(D)** Significant correlation between dSFL and dSR value. ^∗^*P* ≤ 0.05. dSFL, difference value of slope-fascicle length; dSR, difference value of slope-resistance; dSMT, difference value of slope-muscle thickness; dSθ, difference value of slope-phase angle; EIM, electrical impedance myography. Correlation between EIM and ultrasonography parameters.

The two-way ANOVA revealed a significant main effect of the joint angles for the PA (*P* < 0.001) and FL (*P* < 0.001). As ankle plantar flexion increased, the PA value decreased and the FL value increased. MT values only significantly decreased at the angle of 0° and 30°compared with those at −10°. No significant main effect was found in the bilateral comparison of the PA, MT, and FL. No significant interaction effect was indicated (Figure 4). In addition, a significantly lower slope value of the FL angle was found on the affected side than on the unaffected side (*P* = 0.041) (Figure 5).

### Correlation Analysis

**FIGURE 6:**
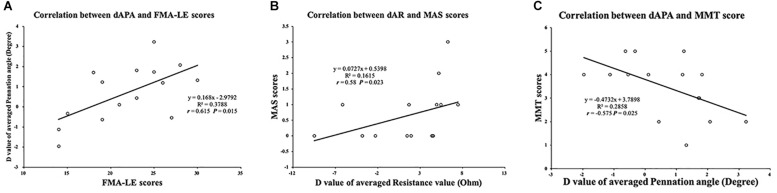
Correlation between muscle intrinsic properties and clinical scales. **(A)** Significant correlation between FMA-LE score and dAPA value. **(B)** Significant correlation between MAS score and dAR value. **(C)** Significant correlation between MMT score and dAPA value. FMA-LE, Fugl–Meyer assessment of lower extremity; MAS, modified ashworth scale; MMT, manual muscle test; dAPA, difference value of average pennation angle parameter; dAR, difference value of average resistance parameter.

Significant differences were found between the affected and unaffected sides in the slope values of θ angle (*P* = 0.003), *R* angle (*P* = 0.005), and *X* angle (*P* = 0.013). The impedance parameters increased more in the unaffected TA muscle when the ankle joint was passively plantar flexed (Figure 5). In terms of the slope value, we correlated the dS value of the EIM and ultrasound parameters between the affected and unaffected TA muscles. Pearson’s correlation analysis revealed a significant correlation between the SMT and dSR (*r* = 0.522, *P* = 0.046), between the dSFL and dSθ (*r* = 0.561, *P* = 0.03), and between the dSFL and dSR (*r* = 0.615, *P* = 0.015) (Figure 5). With regard to the clinical scales, Pearson’s correlation analysis revealed a significant correlation between the dAPA and FMA-LE (*r* = 0.615, *P* = 0.015). Spearman’s correlation coefficients between the dAR and MAS (*r* = 0.58, *P* = 0.023) and between the dAPA and MMT (*r* = −0.575, *P* = 0.025) were statistically significant (Figure 6). No significant correlation was observed between the other parameters.

## Discussion

In this study, ultrasound and EIM measurements were conducted in patients with stroke while the TA muscle was passively stretched through a range of different angles. The results demonstrated changes in the muscle architectural and inherent properties as the ankle joint was stretched. These changes were a decrease in the PA and MT and an increase in the *R*, *X*, and FL. Significant correlations were found between the EIM and ultrasound parameters. This suggested that EIM and ultrasound could alternatively reflect the muscle architectural characteristics.

### Bilateral Difference

Significantly lower *X* and θ values were observed in the affected than in unaffected TA muscle. This outcome was in line with the findings of previous studies that θ could be a sensitive biomarker in the measurement of muscle structure and component properties (Li X. et al., 2017; Zong et al., 2018). However, there was no significant difference in the ultrasound parameters between the affected and unaffected sides. This finding contradicts that of previous studies, which reported a shorter muscle FL in the participants with chronic stroke (Gao et al., 2011). The reduction in the muscle FL was related to the prolonged shortening of the muscle fibers as a consequence of the joint immobilization and spasticity (Ramsay et al., 2014; Thielman, 2019). In the present study, the recruited participants were in the subacute stage of stroke where minimal physiological spasticity was present, as indicated by the MAS scores. During the subacute stage of stroke, flaccid paralysis occurs owing to the loss of neural signal from the cerebral cortex. Muscles become progressively weak, but not to the extent of spasticity. Thus, no significant decrease in muscle FL may occur, as observed in the present study. However, a significant reduction in the EIM parameters in the affected TA muscle was observed in the present study. This finding suggests that EIM may be a more sensitive technique than ultrasonography for the assessment of the muscle inherent properties in the early stage of stroke.

The results of the two-way ANOVA indicated that as the ankle was passively plantarflexed, the FL, *R*, and *X* values of the bilateral TA muscles increased. For the slope values, significantly reduced slope values of FL, *R*, and *X* versus the ROM were revealed in the affected TA muscle. This means that the increasing slope of FL, *R*, and *X* values was lower in the affected than in unaffected TA muscle. The reason that the bilateral TA muscles reacted diversely to the passive stretching might be related to the increase in muscle stiffness and ankle joint spasticity after the occurrence of stroke. It was revealed that the muscle stiffness of the paretic TA muscle estimated from ankle joint kinematics measurements was significantly greater by 23.1% than that of the unimpaired contralateral side (Jakubowski et al., 2017). With the increase in joint stiffness, the altered muscle architecture parameters would respond differently to a passive stretch. The changes in the EIM parameters were in line with the morphological changes and changed in the same way as did the FL value with a lower increasing slope in the paretic TA muscle. The correlation analysis indicated that the changing slope for the ultrasound parameters as the muscle was stretched positively correlated with EIM. Therefore, EIM can be an alternative to detect muscle structure alterations and could provide visualized information and quantitative values. The correlation analysis between the EIM and ultrasonography parameters in the present study verified that the muscle morphology significantly correlated with the muscle impedance properties. Therefore, the EIM and ultrasound data from the current study are helpful for understanding of the mechanism behind the musculoskeletal changes after stroke and can facilitate the evaluation of training effects for patients with stroke.

### Joint-Angle-Dependence Phenomenon

The findings of the present study indicated that as the ankle joint was passively plantarflexed, the muscle FL increased and the PA decreased (Figure 4). This outcome is known as the joint-angle dependence and is in line with the findings of our previous studies (Li et al., 2007; Liu et al., 2014). The TA was the agonist of ankle dorsiflexion, and its myofibers elongate and arrange more horizontally during passive plantar flexion, resulting in a decrease of the PA (Kellis, 2018).

Significant differences were found in the *X* and θ values between the affected and unaffected sides in four joint angles, at 0°, 10°, 20°, and 30° of plantar flexion. This provides strong evidence that EIM could reflect the joint angle change. The two-way ANOVA revealed that there were significant differences in the *X* value at 0°, 10°, 20°, and 30°, whereas no significant difference was found between −10° and 0°. This might be due to the muscle-tendinous length alteration during passive elongation. It was confirmed that during passive stretching of the gastrocnemius muscle, approximately half of the subsequent increase in the muscle-tendinous length was due to elongation of the tendinous structures, and the rest was due to elongation of the muscle fascicles (Loram et al., 2007). However, the elongation of the tendon structures contributes to approximately 60% to 70% of the *in vivo* length (Herbert et al., 2015). To date, there are limited studies on the effect of stretching on the TA muscle structure, and our study only investigated the TA, not the gastrocnemius muscle. The ratio of the range of *in vivo* length where FL initiated to increase might be different from that in the gastrocnemius muscle. However, we can be certain that in the beginning of the muscle passive elongation, the fascicle architecture hardly altered. This might be the potential reason that the *X* value only significantly changed toward the end range of ankle plantar flexion.

In terms of this joint-angle-dependence alteration, our study revealed that the *R* and *X* increased as the ankle joint was passively plantarflexed. As the TA muscle was passively stretched, the muscle fibers lengthened and attenuated. The PA and MT subsequently decreased, which contributed to the reduction in the *X*. The delay in conduction, as reflected by the *X* value, is influenced by the myofiber membrane integrity, tissue interfaces, and non-ionic substances (Ellis et al., 1999; Mulasi et al., 2015). As the PA and MT decrease, the muscle fibers arranged more horizontally, which resulted in reduction in the quantity of myofibers in a given fixed distance between the voltage electrodes. As the joint angle increased, the elongated muscle fibers and myofibers attenuated. As the muscle tissue is nearly incompressible, the passive change in its length must contribute to changes in the fascicle transverse dimensions (van Bavel et al., 1996) and lead to the muscle fibers being arranged more compactly and tightly. Consequently, more myofibers would accumulate in a given cross-sectional area of the muscle, which would lead to an increase in the number of myofiber membranes underneath the voltage electrodes. This subsequently resulted in an increase in the *X* as the joint angle increased.

As for the *R*, it represents the intrinsic resistivity of the skeletal muscle to electric current passing through the extracellular and intracellular fluid (Matthie, 2008). With a given muscular volume, the cross-sectional area of both the muscle bundle and fascicle decreased following the passive stretch. However, CSA negatively correlated with the muscle inherent impedance, and the decrease in the CSA score resulted in an *R* increase (Lukaski, 2013). In addition, the muscle is regarded as a heterogeneous tissue that consists of materials with different levels of conductivity (Matthie, 2008). As the muscle is passively elongated, the intramuscular tissues, such as the myofascial, fat, and connective tissues, are compacted into the myofibers, pushing the extracellular fluid aside. These conductive fluids dropped in the area underneath the two fixed voltage electrodes. Similarly, owing to attenuation of the myofibers, the intracellular fluid in the settled distance between the two voltage electrodes was reduced. This might be another potential reason for the elevation of the *R* value along with ankle joint plantar flexion.

### Clinical Scales

The correlation analysis indicated a significant positive correlation between the averaged *R* values and the MAS score. This finding may be related to the muscle extracellular matrix and non-contractile tissues, such as tendons, ligaments, connective tissue, and other soft tissues, which contributed to the muscle passive stiffness (Chardon et al., 2010; Meyer and Lieber, 2011). In addition, the neural-mediated reflex stiffness varied after stroke, which led to descending influences on the monosynaptic reflex between the muscle spinal afferents and the alpha-motor neurons (Lieber et al., 2017). This is the primary neural mechanism that contributes to muscle tone increase poststroke. This decrease in the variation slope of the *R* values along with the plantar flexion in the paretic TA muscle can possibly be attributed to the ascending muscle tone as well as the non-contractile tissue stiffness after stroke. Hence, in the joint-angle-dependence phenomenon, the slope of the *R* value versus the plantar flexion angle was less than that of the unaffected side.

As for the PA, the increase in PA has two opposite effects. First, the increased PA would allow more muscle fibers and contractile material to attach to the tendon, which would promote force generation (Jones and Rutherford, 1987). Second, according to the muscle force transmitting pattern, as a consequence of the oblique pennate arrangement of the muscle fibers in the TA muscle, the force delivered along the muscle tendon is the product of the force acting along the muscle fibers multiplied by the cosine of the PA (*F*_tendon_ = *F*_fiber_×cos_PA_) (Narici, 1999; Koo et al., 2002). Consequently, a larger PA would result in a less efficient force transmission from the muscle fibers to the tendon (Narici et al., 1996). This contradictory theory of the PA was observed and documented in our study. As the PA decreased in the affected TA muscle, the MMT score of ankle dorsiflexion decreased and the FMA-LE score increased. The reason for the MMT reduction might be that the decreased PA directly brought down the quantity of muscle fibers and contractile material (the dominating effect on muscle force transmitted to the tendon), which ultimately reduced the muscle force generation. In contrast, the decreasing PA of the TA muscle negatively correlated with the FMA-LE score. There might be two possible explanations. First, the decreasing PA elevated the efficiency of force transmission from the fibers to the tendon to a certain extent. From another aspect, the FMA-LE assessed the functional capacity of not only the ankle, but also the knee and hip. Therefore, the correlation between these two parameters provided less clinically meaningful information than that between the PA and the MMT score.

### Limitations

There were some limitations in the current study. First, this was a preliminary study with a sample population of 15 subjects. The recruited sample population included an uneven number of male and female participants, which might contain selection bias. A previous study conducted by Kortman et al. (2013) revealed differences in resistance, reactance, and phase angle between men and women. However, the present pilot study mainly focused on the differences between the affected and the unaffected side, as well as alteration trend of the three parameters versus plantar-flexion angle. In the future study, a large sample population with equal distribution of age, gender, and stage of stroke (i.e., acute, subacute, and chronic) will be recruited. Second, the clinical scales only provided ranked data, which means that significant information might have been lost during the statistical analysis. In a future study, a quantitative assessment of the muscle strength and muscle tone should be taken into consideration, such as using a hand-held dynamometer. The muscle architecture alteration was associated with the muscle function during contraction. Therefore, a future study may include a dynamic assessment of the muscle inherent properties during isometric or isokinetic contraction by means such as electromyography to gain further understanding of the mechanism of muscle alteration after stroke.

## Conclusion

This study demonstrated the feasibility to combine EIM and ultrasonography to assess the muscle architectural and composition alteration in patients in the subacute stage of stroke. There are measurable changes in the TA muscle fascicle impedance and structure, which contribute to the TA muscle dysfunction. The results showed that the impedance changes measured by EIM were associated with the changes in the muscle architectural parameters measured by ultrasonography at different angles of passive joint stretching. Therefore, ultrasonography and EIM could jointly be applied in clinical settings to detect changes in the muscle inherent properties and architectural parameters in patients with subacute stroke. This could assist clinicians to diagnose and evaluate the muscle condition in patients with stroke.

## Data Availability Statement

The datasets analyzed during the current study are available from the corresponding authors upon reasonable request.

## Ethics Statement

The studies involving human participants were reviewed and approved by the Human Subjects Ethics Subcommittee of the First Affiliated Hospital of Sun Yat-sen University. The study was registered on the Chinese Clinical Trial Registry (trial registration number: ChiCTR-IOR-17012299). The patients/participants provided their written informed consent to participate in this study.

## Author Contributions

CH, HH, and LL conceived and designed the study. CH, HH, and XM performed the experiments. CH, HH, and WL wrote the manuscript. WL and LL made a contribution to experiments, and reviewed and edited the manuscript. All authors read and approved the manuscript.

## Conflict of Interest

The authors declare that the research was conducted in the absence of any commercial or financial relationships that could be construed as a potential conflict of interest.
